# Association between psychological resilience and quality of life among middle-aged and older inpatients with chronic diseases: a cross-sectional study

**DOI:** 10.3389/fpubh.2026.1854584

**Published:** 2026-10-20

**Authors:** Jiashuang Xu, Fuzhuo Wang, Yi Chen, Tian Tian, Chenchen Xu, Lin Zhang, Leilei Guo

**Affiliations:** 1 Department of Nursing, Peking Union Medical College Hospital, Chinese Academy of Medical Science and Peking Union Medical College, Beijing, China; 2 Department of General Surgery, The First Affiliated Hospital of Jinzhou Medical University, Jinzhou, Liaoning, China; 3 The Fourth School of Clinical Medicine, China Medical University, Shenyang, Liaoning, China; 4 Department of International Medical, Peking Union Medical College Hospital, Chinese Academy of Medical Science and Peking Union Medical College, Beijing, China; 5 Department of Internal Medicine Nursing, School of Nursing, Wannan Medical College, Wuhu, An Hui Province, China; 6 School of Nursing, Jinzhou Medical University, Jinzhou, Liaoning Province, China

**Keywords:** chronic disease, depression, frailty, psychological resilience, quality of life, successful aging

## Abstract

**Objectives:**

This study aimed to explore the associations between psychological resilience and quality of life in middle- aged and older inpatients with chronic diseases. Additionally, the study investigated the potential mediating role of frailty and depression, as well as the moderating role of successful aging.

**Methods:**

From October 2021 to February 2022, 348 middle-aged and older inpatients with chronic diseases (≥45 years old) were recruited using a multi-stage stratified random sampling method at an affiliated hospital in Zhejiang Province, China. Data were collected using questionnaires and analyzed using SPSS 25.0 and the PROCESS macro. Statistical significance was set at a two-tailed *p* < 0.05.

**Results:**

A total of 348 middle-aged and older inpatients with chronic diseases were included in the analysis. The participants were aged 45–96 years, with a mean age of 63.88 ± 10.49 years; 216 (62.1%) were male and 132 (37.9%) were female. Psychological resilience was significantly associated with quality of life. Three significant mediating pathways were identified: the indirect effect through frailty was 0.0139 (95% CI: 0.0014–0.0300), the indirect effect through depression was 0.0494 (95% CI: 0.0235–0.0809), and the chain indirect effect through frailty and depression was 0.0090 (95% CI: 0.0010–0.0183). Furthermore, successful aging moderated the relationship between frailty and depression.

**Conclusion:**

Psychological resilience was positively associated with quality of life in middle-aged and older inpatients with chronic diseases. Frailty and depression partially mediated this association, while successful aging moderated the relationship between frailty and depression. These findings highlight the potential value of incorporating psychological resilience into comprehensive care for middle-aged and older patients with chronic diseases, together with the early identification and management of frailty and depression. Future longitudinal and intervention studies are warranted to further clarify these pathways and determine whether integrated resilience-, frailty-, and depression-focused interventions can improve quality of life in this population.

## Introduction

With the development of society and population aging, middle-aged and older adults represent a major population affected by chronic diseases (1–3). Chronic diseases are long-term conditions, including cardiovascular diseases, cancers, diabetes, and chronic respiratory diseases, that are characterized by prolonged duration and substantial health burdens (4). Globally, noncommunicable diseases caused at least 43 million deaths in 2021, accounting for approximately 75% of non-pandemic-related deaths (5). As chronic diseases progress, hospitalization may become a common need for affected patients (6). During hospitalization, patients not only face challenges related to disease treatment and rehabilitation but may also experience psychological distress, such as psychological stress, anxiety, and depression, which can adversely affect their quality of life (7–9).

Quality of life is defined by the World Health Organization as individuals’ perceptions of their position in life in the context of the culture and value systems in which they live and in relation to their goals, expectations, standards, and concerns. It is a broad, multidimensional concept that incorporates physical health, psychological state, level of independence, social relationships, personal beliefs, and their relationship to salient features of the environment (10). Many studies have pointed out that improving quality of life is one of the most important ways to improve survival and increase the chances of recovery for inpatients (11, 12). Hence, the main objective of this study was to examine the factors related to quality of life among middle-aged and older inpatients with chronic diseases (MOICDs).

Psychological resilience refers to the capacity to maintain or regain psychological equilibrium under stress or adversity (13). Within Kumpfer’s resilience framework, it is conceptualized not as a fixed trait but as a dynamic process involving the interaction between environmental risk factors, internal resilience factors (cognitive, emotional, physical, behavioral, and spiritual), and adaptation outcomes (14). In this study, resilience was measured using the Connor–Davidson Resilience Scale (CD-RISC), which captures five dimensions: personal competence, tolerance of negative affect, positive acceptance of change, control, and spiritual influence. Higher psychological resilience has been associated with better mental health, social functioning, and quality of life in patients with chronic diseases (15, 16). Resilient individuals are more likely to adopt positive reappraisal and problem-focused coping, which may help preserve quality of life during hospitalization. We therefore hypothesized that psychological resilience would be positively associated with quality of life among MOICDs.

Frailty is a nonspecific condition characterized by elevated vulnerability and decreased stress resistance, which can be manifested as physical, psychological, and social frailty (17). Low psychological resilience may reflect a lack of psychological and social resources, making patients more vulnerable to frailty (18). Frailty, in turn, is an independent risk factor for reduced quality of life among inpatients with chronic diseases, and may be associated with a gradual loss of independence and reduced self-worth (19, 20). We therefore hypothesized that frailty would mediate the relationship between psychological resilience and quality of life among MOICDs.

Depression is marked by substantial and enduring low mood and is common among MOICDs (21). Depression not only worsens prognosis but also reduces immunity, impairs recovery, and lowers quality of life (22). Low psychological resilience is associated with higher levels of anxiety and depression (23), and emotion regulation theory suggests that low resilience may lead to ineffective emotion regulation and increased susceptibility to depression (24). Depression, in turn, reduces health motivation and self-care behaviors, further lowering quality of life (25). We therefore hypothesized that depression would mediate the relationship between psychological resilience and quality of life among MOICDs.

Based on the biopsychosocial model, frailty is recognized as a risk factor for depression (26). Frailty can trigger adverse events such as falls, pain, and social isolation, which may exacerbate depression (27, 28). Depression can then limit mental activity, reduce interest, and impair self-care, ultimately lowering quality of life (29). Stress-adaptation theory further suggests that psychological resilience reflects positive adaptation, whereas frailty and depression represent negative stress reactions, with quality of life as the outcome. We therefore hypothesized that frailty and depression would sequentially mediate the relationship between psychological resilience and quality of life among MOICDs.

Successful aging can help slow functional decline, reduce the progression of chronic diseases, and mitigate the adverse consequences of frailty (30). It also helps patients develop a positive mindset and reduces anxiety and depression (31). The ecological theory of aging suggests that successfully aging individuals can better utilize internal coping resources and external social resources, buffering the effect of frailty on depression (32, 33). However, evidence on this moderating role in MOICDs remains insufficient. We therefore hypothesized that successful aging would moderate the relationship between frailty and depression, such that the positive association between frailty and depression would be weaker among those with higher levels of successful aging.

In summary, frailty and depression may act as chain mediators between psychological resilience and quality of life, while successful aging may moderate the frailty-depression link. This study proposes five hypotheses: Hypothesis 1 psychological resilience is positively associated with quality of life; Hypothesis 2 frailty mediates this relationship; Hypothesis 3 depression mediates this relationship; Hypothesis 4 frailty and depression sequentially mediate this relationship; and Hypothesis 5 successful aging moderates the frailty-depression link. This study aims to test this moderated chain mediation model (Figure 1), providing theoretical and empirical evidence for improving the quality of life of MOICDs.

**Figure 1:**
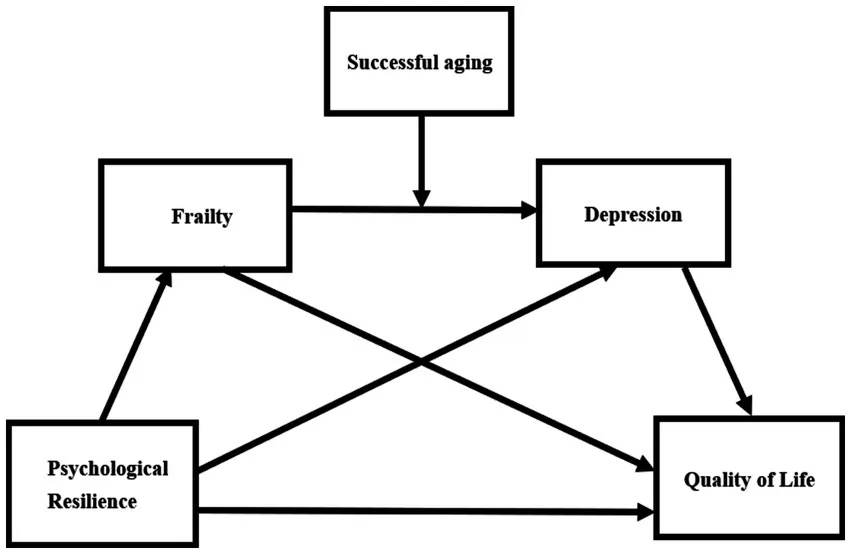
Hypothetical model.

## Methods

### Participants and data collection

A cross-sectional design was used in this study. Data collection was conducted through a multi-stage stratified sampling method between October 2021 and February 2022 in Shangcheng District, Hangzhou, Zhejiang Province. Random numbers were used to determine the sampling units. First, one tertiary hospital in the study area was randomly selected. Second, departments within the selected hospital were randomly selected. Third, middle-aged and older inpatients with chronic diseases who met the inclusion criteria were randomly selected from the survey stations established in the selected departments. Table 1 provides a detailed list of the specific types of chronic diseases represented in the sample. Ultimately, a face-to-face survey was administered to 348 MOICDs who met the inclusion criteria.

**Table 1:** Prevalence of diseases in various organ systems.

Variables	*N* (%)
1. Heart	100 (28.7)
2. Blood vessels	151 (43.4)
3. Hematopoietic system (bone marrow)	42 (12.1)
4. Respiratory	88 (25.3)
5. Eye, ear, nose and throat	38 (10.9)
6. Upper gastrointestinal tract	51 (14.7)
7. Lower gastrointestinal tract	65 (18.7)
8. Liver	112 (32.2)
9. Kidney	54 (15.5)
10. Urogenital	50 (14.4)
11. Motor system	98 (28.2)
12. Nervous system	89 (25.6)
13. Endocrine/metabolic and breast cancer	103 (29.6)
14. Psychiatric	0 (0.0)

The sample size was estimated using the Kendall sample size rough approximation method (34), which suggests that the sample size should be 5 to 10 times the number of variables, with an additional 20% allowance for potential sample attrition. Specifically, 29 variables were included in this study, including 7 demographic variables, 1 chronic disease dimension, 5 psychological resilience dimensions, 3 frailty dimensions, 1 depression dimension, 4 successful aging dimensions, and 8 quality of life dimensions. Therefore, according to our preliminary sample size estimation range: the minimum sample size: is 5 × 29 = 145 (not considering attrition); the maximum sample size: is 10 × 29 = 290 (not considering attrition). After allowing for 20% sample attrition, the required number of valid samples was estimated to range from 181 (145 ÷ 0.8) to 363 (290 ÷ 0.8). During the data collection process, the research team distributed a total of 355 questionnaires to obtain a sufficient number of valid responses. In the end, 348 valid questionnaires were collected, with an effective response rate of 98.03%.

This sample size exceeded the minimum required number of valid samples and provided an adequate number of participants for the planned analyses. Before the survey, 17 investigators underwent standardized training to improve their communication skills and ensure a consistent understanding of the scoring procedures. Inclusion criteria: (i) willingness to participate in the study; (ii) clear consciousness and no obstacle to verbal communication; (iii) age ≥ 45 years. Exclusion criteria: (1) completion of less than 50% of the questionnaire. (2) withdrawal from the study before completion. (3) severe psychiatric disorders that prevented effective communication (35). After informed consent was given, eligible patients with chronic diseases received questionnaires and completed them under one-on-one guidance from trained researchers; patients then completed the questionnaires. All study procedures adhered to the principles outlined in the Declaration of Helsinki.

### Demographic characteristics

The demographic characteristics encompass age, gender, marital status, economic status, physical activity, social activity, and chronic pain.

### Cumulative illness rating scale

The Cumulative Illness Rating Scale (CIRS) was developed by Linn et al. in 1968 (36) and has been widely used in middle-aged and older patients with chronic diseases (37). The scale consists of 14 items, each corresponding to an organ system; for example, the “Respiratory” item covers diseases such as chronic obstructive pulmonary disease (COPD), asthma, or pneumonia. In this study, the CIRS was used only to identify the presence or absence of disease in each organ system rather than to grade disease severity. Each system was coded as 0 (no disease) or 1 (disease present), and the number of affected organ systems was used to describe the participants’ comorbidity burden. This approach provided a comprehensive overview of the participants’ health status.

### Psychological resilience scale

The Psychological Resilience Scale was developed by American psychologists Connor and Davidson in 2003 based on a research program on post-traumatic stress disorder, and it has been widely used to assess the level of psychological resilience in the general population and clinical patients (38, 39). The scale consists of 25 entries organized into five dimensions: personal competence (e.g., “I am proud of my accomplishments”), tolerance of negative feelings (e.g., “I can handle unhappy emotions”), positive acceptance of the change (e.g.: “I can make unusual or difficult decisions”), control (e.g., ‘I know where to go for help’), and spiritual influence (e.g.,” Sometimes, fate or God can help “). A 5-point scale (0–4) was adopted with a total score ranging from 0 to 100, with higher scores indicating greater psychological resilience. In this analysis, the Cronbach’s alpha coefficient for the scale was 0.932, and the KMO value was 0.868.

### Frailty scale

The Tilburg Frailty Scale was developed by Dutch researchers in 2010 to assess an individual’s level of frailty and is now widely used (40, 41). The scale has now been validated in several studies, demonstrating its ability to effectively differentiate between individuals with different levels of frailty and its high clinical applicability (42, 43). The scale consists of three dimensions: somatic frailty (e.g., “Do you feel physically healthy?”), psychological frailty (e.g., “Have you felt nervous or anxious in the last month?”) (e.g., “Do you miss the company of friends and family?”). The scale consists of 15 entries with a score range of 0 to 15, with scores of 5 and above indicating frailty, and higher scores indicating more severe frailty. The Cronbach’s coefficient for this scale in this study was 0.716 and the KMO was 0.650.

### Depression scale

The Patient Health Questionnaire-9 (PHQ-9) was created by Spitzer in 1999 to assess depression in individuals (44). The scale has been validated in middle-aged and older patients with chronic diseases and has good reliability and validity (45). It consists of 9 items with a single dimension (e.g., thoughts of dying or hurting oneself in some way). A 4-point rating system is used, with scores for each item ranging from 0 (not at all) to 3 (almost every day) and total scores ranging from 0 to 27. Depending on the total score, the degree of depressive symptoms can be categorized into the following stages: “0–4” indicates no depression, “5–9” indicates depression, “10–14” indicates significant depression, and “15” and above indicates severe depression. Here, the Cronbach’s coefficient for this scale was 0.836 and the KMO was 0.866.

### Successful aging scale

The Successful Aging Inventory (SAI) was developed by Lee et al. in 2017 (46). The SAI scale has been validated in several studies, which have shown that it has good structural and content validity and that it accurately reflects an individual’s adjustment to the aging process and level of successful aging (39, 47). The scale consists of 16 items covering four dimensions: active participation (e.g., “Continued learning”), health resources (e.g., “Being able to think well/clearly “), positive spirituality (e.g., “Positive attitudes”), and valued relationships (e.g., “Good relationship with family”). The scale utilizes a four-point Likert scale ranging from 1 (“not at all important”) to 4 (“very important”). The total score ranges from 16 to 64, with higher scores indicating more successful aging. In this study, the Cronbach’s coefficient for the scale was 0.827 and the KMO was 0.811.

### Quality of life scale

The SF-12 is a derived instrument consisting of 12 items extracted from the SF-36 questionnaire, developed by the Boston Medical Center in the United States in 1996 (48). The SF-12 scale has been extensively used in a variety of populations and has been validated in several studies with good construct and content validity (49, 50). The scale consists of 12 entries divided into 8 dimensions: somatic activity functioning (e.g., “moderate-intensity activities, such as moving a table, playing tai chi, etc.”), somatic role functioning (e.g., “so that you can only partially accomplish what you want to do”), organic pain (e.g., “In the past four weeks, to what extent have pain in your body prevented you from going about your daily life, outdoor activities, or household chores?”), general health status (e.g., “Overall, how do you think your health is now?”), vitality (e.g., “Feeling energetic?”), social functioning (e.g., “Has your socializing been affected by health or emotional problems?”), emotional functioning (e.g., “making what you want to do only partially accomplished”), and psychological functioning (e.g., “feeling at peace?”). The scale scores range from 0 to 100 points, with higher scores reflecting better quality of life. In this research, the scale had a Cronbach’s coefficient of 0.860 and a KMO of 0.835.

### Statistical analysis

For data analysis, IBM SPSS Statistics 25.0 was used. Descriptive statistics were presented as frequencies, percentages, means, and standard deviations. Independent *t*-tests and one-way ANOVAs were used to compare quality-of-life scores across demographic characteristics. Pearson correlation analyses were conducted to examine the associations among psychological resilience, frailty, depression, successful aging, and quality of life. Given the exploratory nature of these analyses, no corrections for multiple comparisons were applied (51). These analyses were conducted primarily to describe sample characteristics and identify variables potentially associated with quality of life, rather than to test independent hypotheses. Based on these preliminary analyses and their clinical relevance to quality of life, age, physical activity, social activity, and chronic pain were included as covariates in the subsequent mediation and moderated mediation analyses. Before the mediation and moderated mediation analyses, the assumptions of linear regression were examined. Linearity and homoscedasticity were assessed using residual-versus-fitted plots. Multicollinearity was evaluated using the variance inflation factor (VIF) and tolerance values (all VIF < 5, all tolerance > 0.20). Residual normality was examined using normal P–P plots, and independence of residuals was assessed using the Durbin–Watson statistic. All diagnostic results indicated that the assumptions were adequately met. The PROCESS 3.5 macro was then used to test the mediation and moderated mediation models. Model 6 was used to examine whether frailty and depression mediated the relationship between psychological resilience and quality of life. Model 91 was used to test whether successful aging moderated the frailty → depression pathway, and a simple slope test was used to probe the moderating effect at low, medium, and high levels of successful aging. For all analyses, 5,000 bootstrap resamples were used to calculate 95% bias-corrected confidence intervals (CIs); effects were considered significant when the 95% CI did not include zero.

## Results

### Descriptive statistics

Table 2 displays the demographic characteristics and findings of the univariate analysis. The distribution of chronic disease types among the study participants is presented in Table 1. In our research, the age range of the study participants was 45–96 years, with an average age of 63.88 ± 10.49 years. A total of 216 (62.1%) participants were male and 132 (37.9%) were female. Most of the study respondents were married (98.0%), and approximately 45.4% reported having a balanced income. In addition, the univariate analysis showed that age, physical activity, social activity, and chronic pain were significantly associated with quality of life (*p* < 0.05). These variables were therefore included as covariates in the subsequent mediation and moderated mediation analyses.

**Table 2:** Univariate analysis of quality of life among inpatients with different characteristics of chronic diseases (N = 348).

Variables	Group	*N* (%)	Mean ± SD	*F*/*t*	*p*
Age	45–64	178 (51.1)	77.10 ± 10.02	9.318	0.002
	≥65	170 (48.9)	73.83 ± 9.96		
Sex	Male	216 (62.1)	75.32 ± 9.49	0.180	0.671
	Female	132 (37.9)	75.81 ± 11.07		
Marital status	Married	341 (98.0)	75.50 ± 10.16	0.010	0.922
	Unmarried	7 (2.0)	75.87 ± 7.86		
Economic status	Balance available	145 (41.7)	75.97 ± 9.93	0.394	0.675
	Balance of income and expenditure	158 (45.4)	74.98 ± 10.04		
	Income not enough to cover expenses	45 (12.9)	75.85 ± 11.06		
Physical activity	Regular activity	92 (26.4)	79.33 ± 9.43	17.085	<0.001
	Irregular activity	208 (59.8)	75.24 ± 9.58		
	Inactive	48 (13.8)	69.32 ± 10.46		
Social activity	non-participation	66 (19.0)	71.94 ± 9.71	11.276	<0.001
	Occasional participation	210 (60.3)	75.16 ± 9.97		
	Frequent participation	72 (20.7)	79.77 ± 9.46		
Chronic pain	Yes	109 (31.3)	71.69 ± 8.94	24.159	<0.001
	No	239 (68.7)	77.25 ± 10.15		

### Correlations between overall variables

Pearson’s correlation analysis showed that psychological resilience was negatively associated with frailty and depression (*r* = −0.146, *p* < 0.01; *r* = −0.323, *p* < 0.01) and positively associated with successful aging and quality of life (*r* = 0.347, *p* < 0.01; *r* = 0.383, *p* < 0.01). Frailty was positively associated with depression (*r* = 0.356, *p* < 0.01) and negatively associated with quality of life (*r* = −0.340, *p* < 0.01). Depression was negatively associated with successful aging and quality of life (*r* = −0.196, *p* < 0.01; *r* = −0.493, *p* < 0.01). Although successful aging was not significantly correlated with quality of life (*r* = 0.026, *p* > 0.05), this did not preclude its role as a moderator, because a moderator is evaluated based on whether it changes the strength of the association between a predictor and an outcome rather than on its bivariate correlation with the outcome. See Table 3.

**Table 3:** Correlation analysis

Variables	Mean	SD	1	2	3	4	5
1. Psychological Resilience	49.88	13.86	1				
2. Frailty	6.31	2.19	−0.146^**^	1			
3. Depression	7.04	4.39	−0.323^**^	0.356^**^	1		
4. Successful aging	51.34	7.35	0.347^**^	0.074	−0.196^**^	1	
5. Quality of Life	75.50	10.11	0.383^**^	−0.340^**^	−0.493^**^	0.026	1

^**^p < 0.01.

### Mediation analysis

Model 6 of the PROCESS macro (version 3.5) developed by Hayes was used to test Hypotheses 1–4 as stated in the Introduction. In this model, X denotes psychological resilience, M1 denotes frailty, M2 denotes depression, and Y denotes quality of life.

The results showed a significant positive association between psychological resilience and quality of life (*B* = 0.146, *p* < 0.001); therefore, Hypothesis 1 was supported. Psychological resilience was negatively associated with frailty (*B* = −0.020, *p* < 0.05) and depression (*B* = −0.070, *p* < 0.001). Frailty was positively associated with depression (*B* = 0.652, *p* < 0.001) and negatively associated with quality of life (*B* = −0.704, *p* < 0.001). Depression was negatively associated with quality of life (*B* = −0.705, *p* < 0.001) (see Table 4).

**Table 4:** Regression analysis of the mediation model.

Predictors	Quality of Life				Frailty				Depression				Quality of Life			
	*B*	SE	*t*	95%CI	*B*	SE	*t*	95%CI	*B*	SE	*t*	95%CI	*B*	SE	*t*	95%CI
Age	−2.206	0.971	−2.273	(−4.115,0.297)	1.036	0.232	4.471^***^	(0.580,1.492)	−0.243	0.433	−0.562	(−1.094, 0.608)	−1.171	0.913	−1.284	(−2.967, 0.624)
Physical activity	−2.968	0.785	−3.783^***^	(−4.512, −1.425)	−0.281	0.187	−1.502	(−0.650,0.087)	1.633	0.341	4.788^***^	(0.962, 2.303)	−2.145	0.743	−2.887^**^	(−3.606, −0.683)
Social activity	2.008	0.787	2.553^*^	(0.461,3.555)	−0.234	0.188	−1.247	(−0.604,0.135)	0.614	0.341	1.797	(−0.058, 1.285)	2.168	0.724	2.996^**^	(0.745, 3.591)
Chronic pain	4.028	1.029	3.915^***^	(2.004,6.052)	−0.667	0.246	−2.715^**^	(−1.150, −0.184)	−1.192	0.451	−2.646^*^	(−2.078,- 0.306)	2.412	0.960	2.512^*^	(0.524, 4.300)
Psychological Resilience	0.218	0.035	6.225^***^	(0.149, 0.287)	−0.020	0.008	−2.357^*^	(−0.036, −0.003)	−0.070	0.015	−4.585^***^	(−0.100, −0.040)	0.146	0.033	4.382^***^	(0.080, 0.211)
Frailty									0.652	0.098	6.643^***^	(0.459, 0.845)	−0.704	0.220	−3.204^**^	(−1.137, −0.272)
Depression													−0.705	0.114	−6.173 ^***^	(−0.930, −0.480)
*R*	0.514				0.326				0.521				0.624			
*R^2^*	0.265				0.107				0.272				0.390			
*F*	24.600 ^***^				8.185				21.198 ^***^				31.015			

^*^p < 0.05, ^**^p < 0.01, ^***^p < 0.001.

The mediation analysis showed that frailty significantly mediated the association between psychological resilience and quality of life (indirect effect = 0.0139, 95% CI [0.0014, 0.0300]), accounting for 6.38% of the total effect; therefore, Hypothesis 2 was supported. Depression also significantly mediated this association (indirect effect = 0.0494, 95% CI [0.0235, 0.0809]), accounting for 22.68% of the total effect; therefore, Hypothesis 3 was supported. In addition, the sequential indirect effect through frailty and depression was significant (indirect effect = 0.0090, 95% CI [0.0010, 0.0183]), accounting for 4.14% of the total effect; therefore, Hypothesis 4 was supported. The total indirect effect was 0.0723 (95% CI [0.0399, 0.1305]), accounting for 33.20% of the total effect. The total effect of psychological resilience on quality of life was significant (*B* = 0.2178, 95% CI [0.1490, 0.2866]). The direct effect of psychological resilience on quality of life remained significant (*B* = 0.1455, 95% CI [0.0802, 0.2108]), indicating partial mediation, as the direct effect remained significant after accounting for frailty and depression (see Table 5).

**Table 5:** Mediation effects test.

Path	Effect Value	S.E.	95% CI		Effectiveness ratio %
			LLCI	ULCI	
Direct effect					
X → Y	0.1455	0.0332	0.0802	0.2108	66.80
Indirect effect					
X → M1 → Y	0.0139	0.0073	0.0014	0.0300	6.38
X → M2 → Y	0.0494	0.0149	0.0235	0.0809	22.68
X → M1 → M2 → Y	0.0090	0.0044	0.0010	0.0183	4.14
Total Indirect effect	0.0723	0.0179	0.0399	0.1305	33.20
Total effect	0.2178	0.0350	0.1490	0.2866	100

*X* is Psychological Resilience, M1 is Frailty, M2 is Depression, and Y is Quality of Life.

These results indicate that the association between psychological resilience and quality of life was partially mediated by frailty and depression, both independently and sequentially. The largest indirect contribution was through depression alone, followed by frailty alone, and then the sequential frailty–depression pathway.

### Moderated mediation analysis

Model 91 of the PROCESS macro was used to test Hypothesis 5, that is, whether successful aging moderated the frailty → depression pathway in the mediated model. As shown in Table 6, frailty had a significant main effect on depression (*B* = 0.687, SE = 0.096, 95% CI [0.497, 0.877]), whereas successful aging significantly moderated this association (*B* = −0.045, SE = 0.014, 95% CI [−0.072, −0.017]). Therefore, Hypothesis 5 was supported. The final model is presented in Figure 2.

**Table 6:** Results of the moderated mediation model analysis.

Predictors	Frailty (Model 1)				Depression (Model 2)				Quality of life (Model 3)			
	*B*	SE	*t*	95%CI	*B*	SE	*t*	95%CI	*B*	SE	*t*	95%CI
Age	1.036	0.231	4.472^***^	(0.580,1.492)	−0.116	0.426	−0.273	(−0.954, 0.721)	−1.171	0.913	−1.284	(2.967, 0.624)
Physical activity	−0.281	0.187	−1.502	(−0.650, 0.087)	1.624	0.334	4.868^***^	(0.968, 2.279)	−2.145	0.743	- 2.887^**^	(−3.606, −0.683)
Social activity	−0.234	0.188	−1.247	(−0.604, 0.135)	0.681	0.334	2.039^*^	(0.024, 1.338)	2.168	0.724	2.996^**^	(0.745, 3.591)
Chronic pain	−0.667	0.246	−2.715^**^	(−1.150, −0.184)	−0.980	0.442	−2.258^*^	(−1.867, −0.129)	2.412	0.961	2.512^*^	(0.524, 4.300)
Psychological Resilience	−0.020	0.008	−2.356^*^	(−0.036, −0.003)	−0.059	0.016	−3.674^***^	(−0.090, −0.027)	0.146	0.033	4.382^***^	(0.080, 0.211)
Frailty					0.687	0.096	7.126^***^	(0.497, 0.877)	−0.704	0.220	−3.204^**^	(−1.137, −0.272)
Successful aging					−0.093	0.030	−3.146^**^	(−0.151, −0.035)				
Frailty *Successful aging					−0.045	0.014	−3.216^**^	(−0.072, −0.017)				
Depression									−0.705	0.114	−6.173^***^	(−0.928, −0.480)
*R*	0.327				0.557				0.624			
*R^2^*	0.107				0.310				0.390			
*F*	8.185^***^				19.064^***^				31.015^***^			

^*^p < 0.05, ^**^p < 0.01, ^***^p < 0.001.

**Figure 2:**
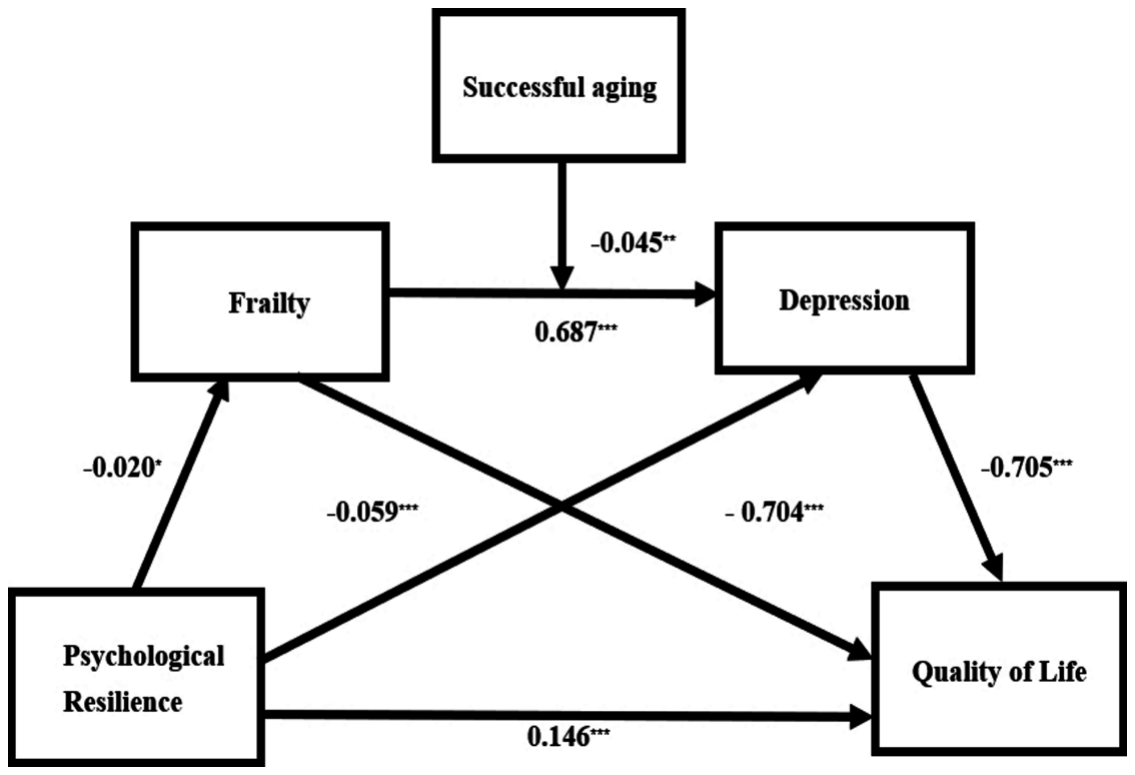
Verified moderated chain mediation model.

To further interpret the moderating effect of successful aging, successful aging was categorized into three levels: low (one standard deviation below the mean), moderate (at the mean), and high (one standard deviation above the mean). As shown in Table 7 and Figure 3, frailty was a significant positive predictor of depression among participants with low levels of successful aging (*B* = 1.016, SE = 0.140, t = 7.256, *p* < 0.001). However, this positive association was attenuated among participants with high levels of successful aging (B = 0.359, SE = 0.141, t = 2.553, *p* < 0.05). These findings indicate that successful aging buffered the positive association between frailty and depression.

**Table 7:** Moderating effects of different levels of successful aging.

Successful aging	Effect	Boot SE	95% CI	
			Boot LLCI	Boot ULCI
M − 1SD	1.016	0.140	0.785	1.247
M	0.688	0.096	0.530	0.846
M + 1SD	0.359	0.141	0.127	0.591

**Figure 3:**
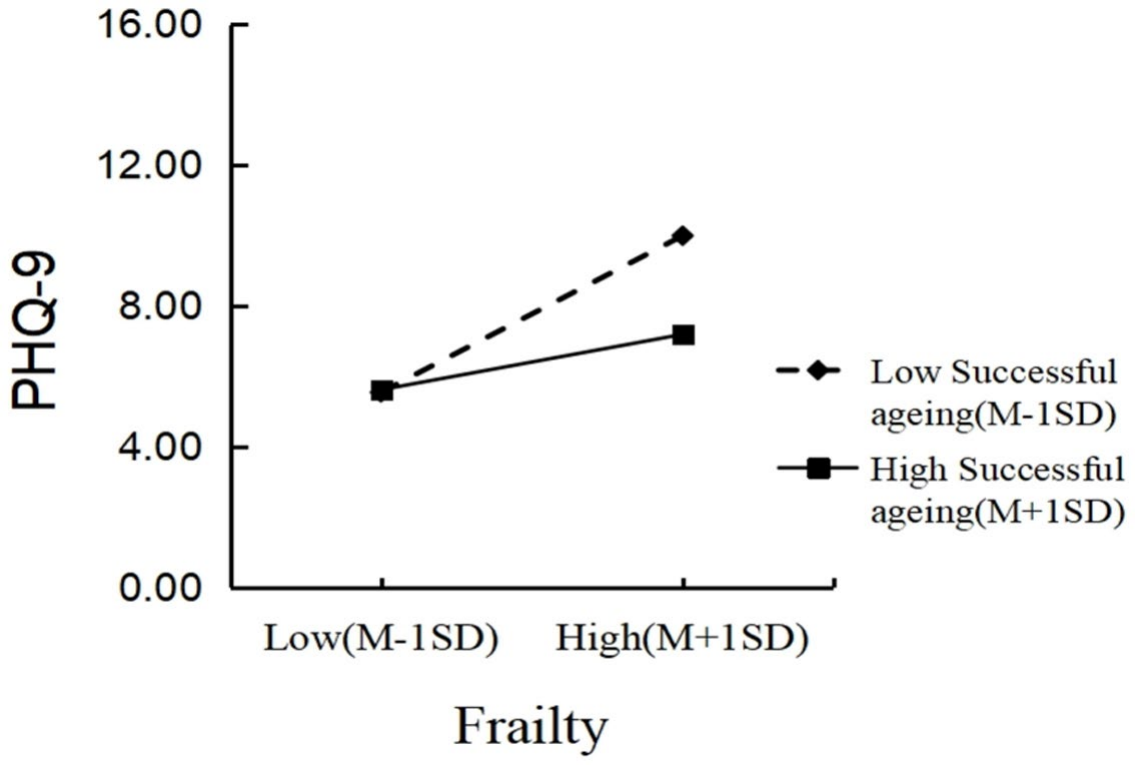
Plot of the relationship between frailty and depression at two levels of successful aging.

### Hypothesis testing summary

In summary, Hypotheses 1–5 were all supported. Psychological resilience was positively associated with quality of life, and this association was partially mediated by frailty and depression, both independently and sequentially. In addition, successful aging significantly attenuated the positive association between frailty and depression.

## Discussion

The current study found a positive relationship between psychological resilience and quality of life in MOICDs, which is consistent with past research findings and with the theoretical perspective of the psychological resilience framework (52–54). High levels of psychological resilience have been associated with better cognitive functioning among MOICDs, which may be related to more positive attitudes toward disease and treatment and, in turn, better quality of life (55). On the contrary, MOICDs with low psychological resilience may lack positive cognition and the ability to effectively cope with difficulties in coping with various stresses and changes in the course of their diseases and treatments. These patients may be more likely to experience negative thinking patterns, which may be associated with less positive attitudes toward treatment and lower quality of life (56). Hence, future intervention studies could examine whether enhancing psychological resilience is associated with improved quality of life among MOICDs.

The results of this study indicate that frailty mediates the relationship between psychological resilience and quality of life among MOICDs, which is consistent with prior findings and in line with the demand-resource model (57). Specifically, prolonged treatment may be associated with lower self-efficacy among MOICDs, referring to their confidence in effectively coping with disease-related difficulties, which may in turn be associated with lower psychological resilience. Low levels of psychological resilience may reflect fewer psychological and social resources and may be associated with greater difficulties in coping with disease and treatment demands, which may in turn be related to greater physical and psychological burdens and higher levels of frailty (18). The initiation and progression of frailty may be associated with lower independence, self-esteem, and sense of self-worth among MOICDs, which may in turn be associated with lower quality of life (58). Thus, in addition to improving psychological resilience, we recognize that attention should be paid to the frailty of MOICDs and timely, targeted measures should be taken to improve the degree of frailty, which may help improve the overall quality of life of MOICDs.

The present study found that depression mediated the relationship between psychological resilience and quality of life among MOICDs, which is consistent with previous findings (59). Emotion regulation theory suggests that low psychological resilience may be associated with a lack of effective emotion regulation strategies and adaptive coping styles among MOICDs when facing stress and frustration. This deficiency may be associated with greater susceptibility to negative emotions and higher levels of depressive symptoms (60, 61). Depression may be associated with reduced attention to health status and lower motivation toward healthcare plans and rehabilitation processes, which may in turn be associated with poorer health-related outcomes and lower quality of life (62). Therefore, paying attention to depression trends in MOICDs and timely interventions to alleviate depression appear to be crucial for improving their quality of life.

Our findings suggest that frailty and depression act as chain mediators between psychological resilience and quality of life in MOICDs. This result can be better explained by stress-adaptation theory (63). In this theory, chronic diseases (especially acute exacerbations) are viewed as stressors, psychological resilience represents positive adaptation, frailty and depression represent negative stress reactions reflecting maladaptation, and the high or low quality of life of MOICDs is viewed as an outcome resulting from stress reactions. During hospitalization, patients with chronic diseases may need to undergo various therapeutic measures such as medication, surgery, and radiotherapy, which may be associated with physical discomfort and psychological burden and may also be related to psychological resilience (64). MOICDs with lower psychological resilience may be more likely to experience adverse emotions such as anxiety, depression, and fear, which may be associated with differences in immune, nervous, and endocrine functioning and with higher levels of physical, psychological, and social frailty (65). Frailty may be associated with greater physical discomfort, including pain and fatigue, as well as a greater sense of loss of control over one’s body. This sense of loss of control may also be associated with poorer psychological wellbeing and higher levels of depressive symptoms (66). Depression is often accompanied by sleep problems, including difficulty falling asleep, decreased sleep quality, and early awakenings. These problems can further reduce the energy and vitality of MOICDs and affect their quality of life (67). Consequently, the results of this study support the stress-adaptation theory and emphasize the importance of increasing psychological resilience, improving frailty, and preventing depression to improve the quality of life of MOICDs.

Additionally, our study found that successful aging produced a moderating effect between frailty and depression. To be specific, we observed a significant positive predictive effect of frailty on depression among MOICDs with lower levels of successful aging, whereas the positive predictive effect of frailty on depression was attenuated among MOICDs with higher levels of successful aging. Aging theory (68) suggests that MOICDs who age successfully typically have more positive resources on physical, psychological, and social levels, such as cognitive flexibility and social support. These resources may be associated with greater resilience and more positive coping with disease-related challenges and changes, which may help explain the weaker association between frailty and depression observed among participants with higher levels of successful aging. However, we also need to recognize that the aging process can be influenced by values, biological limitations, and social circumstances. Therefore, a more holistic approach to the lives of MOICDs may be warranted, with attention to support and resources that are associated with successful aging and the observed relationship between frailty and depression. This finding provides positive insights for clinical practice. In treatment and care, healthcare professionals may consider approaches that support successful aging, while further research is needed to determine whether such approaches are associated with lower frailty and better mental health (69, 70). Hence, higher levels of successful aging were associated with a weaker positive association between frailty and depression among MOICDs.

## Limitations

This research has several limitations. Firstly, it employed a cross-sectional design, which does not capture the dynamic changes in psychological resilience, frailty, depression, successful aging, and quality of life among MOICDs and precludes conclusions about causal relationships. Future studies could use longitudinal designs to better understand the long-term relationships among these factors. Secondly, the data relied primarily on patient self-reports, which may have introduced recall and subjective evaluation bias. In addition, regarding covariate selection, economic status was assessed using a single self-reported item, and educational attainment was not collected in the original survey. Although age, physical activity, social activity, and chronic pain were included as covariates based on their preliminary associations with quality of life and clinical relevance, bivariate associations alone do not establish confounding. Therefore, residual confounding related to socioeconomic status, educational attainment, and other unmeasured factors cannot be completely excluded. Future research could incorporate objective measures and third-party assessments to reduce self-report bias, and should also include more comprehensive indicators of socioeconomic status and educational attainment. Finally, the representativeness of the sample may be limited because only one tertiary hospital was selected, and differences in patient distribution across departments and hospitalization duration may have affected the sample. Future studies should recruit participants from multiple hospitals and departments to improve sample representativeness and external validity.

## Implications

At the theoretical level, this study developed a moderated chain mediation model that provides insight into the relationships among psychological resilience, frailty, depression, successful aging, and quality of life. At the practical level, the findings suggest that psychological resilience, frailty, and depression may be relevant considerations in efforts to understand and address quality of life among MOICDs. These may include psychological resilience training, emotion management, appropriate medical care, and social support (71–74). In addition, our findings suggest that successful aging significantly moderates the association between frailty and depression. Therefore, healthcare professionals may consider approaches such as cognitive training, disease management, and mental health support that may support successful aging, while longitudinal and intervention studies are needed to determine whether these approaches alter the association between frailty and depression (75–77).

## Conclusion

The present study examined a moderated chain mediation model and found preliminary evidence that psychological resilience was associated with the quality of life of MOICDs through frailty and depression. Furthermore, successful aging moderated the association between frailty and depression within the chain mediation pathway of “psychological resilience → frailty → depression → quality of life.” These findings hold theoretical and practical value for researchers and healthcare professionals dedicated to improving the quality of life for MOICDs.

## Data Availability

The raw data supporting the conclusions of this article will be made available by the authors, without undue reservation.

## References

[1] Li L Meng J Wu Y Bu X Gao L Xiao Z . Association of grip strength, sleep duration, and comorbidities with depressive symptoms in middle-aged and older patients with chronic lung diseases: a cross-sectional network analysis based on CHARLS data. Front Psychol. (2024) 15:1472766. doi: 10.3389/fpsyg.2024.1472766, 39664637 PMC11632308

[2] Pourrazavi S Bazargan-Hejazi S. The model of online health information-seeking behavior among older adults. BioSocial Health J. (2024) 1:33–40. doi: 10.34172/bshj.1

[3] Adelirad F Javadivala Z Dianat I Amrahi Tabieh A Chattu VK. Physical and cognitive-based training in healthy older adults: evidence from a systematic review and meta-analysis. BioSocial Health J. (2024) 1:14–25. doi: 10.34172/bshj.3

[4] Nugent R. Preventing and managing chronic diseases. BMJ (Clin Res ed). (2019) 364:l459. doi: 10.1136/bmj.l459, 30705038

[5] Castilho EA Goldbaum M. Chronic noncommunicable diseases and population surveys. Rev Saude Publica. (2017) 51:1s. doi: 10.1590/s1518-8787.201705100supl1ed, 28591360 PMC5676357

[6] Bielemann RM Silva BG Coll CD Xavier MO Silva SG. Burden of physical inactivity and hospitalization costs due to chronic diseases. Rev Saude Publica. (2015) 49:75. doi: 10.1590/S0034-8910.2015049005650 PMC4603264 26487291

[7] Billett MC Campanharo CRV Lopes M Batista REA Belasco AGS Okuno MFP. Functional capacity and quality of life of hospitalized octogenarians. Rev Bras Enferm. (2019) 72:43–8. doi: 10.1590/0034-7167-2017-0781, 31826190

[8] Kendir C van den Berg M Guanais F Valderas JM Klazinga N. Lessons from early implementation of the OECD’S patient-reported Indicator surveys (PaRIS) in primary care: making the case for co-development and adaptation to national contexts. Int J Qual Health Care Commun. (2023) 3:lyad003. doi: 10.1093/ijcoms/lyad003

[9] de Boer D van den Berg M Ballester M Bloemeke J Boerma W de Bienassis K . Assessing the outcomes and experiences of care from the perspective of people living with chronic conditions, to support countries in developing people-centred policies and practices: study protocol of the international survey of people living with chronic conditions (PaRIS survey). BMJ Open. (2022) 12:e061424. doi: 10.1136/bmjopen-2022-061424, 36123114 PMC9486339

[10] WHOQOL Group. The World Health Organization quality of life assessment (WHOQOL): position paper from the World Health Organization. Soc Sci Med. (1995) 41:1403–9. doi: 10.1016/0277-9536(95)00112-K, 8560308

[11] Hoekstra T Jaarsma T van Veldhuisen DJ Hillege HL Sanderman R Lesman-Leegte I. Quality of life and survival in patients with heart failure. Eur J Heart Fail. (2013) 15:94–102. doi: 10.1093/eurjhf/hfs148, 22989869

[12] Nasser Albarqi M. Continuity and sustainability of care in family medicine: assessing its association with quality of life and health outcomes in older populations-a systematic review. PLoS One. (2024) 19:e0299283. doi: 10.1371/journal.pone.0299283, 39715241 PMC11666006

[13] Zábó V Csiszar A Ungvari Z Purebl G. Psychological resilience and competence: key promoters of successful aging and flourishing in late life. GeroScience. (2023) 45:3045–58. doi: 10.1007/s11357-023-00856-9, 37418098 PMC10643728

[14] Kumpfer KL. Factors and processes contributing to resilience: the resilience framework. In: Glantz MD, Johnson JL, editors. Resilience and Development: Positive Life Adaptations. New York, NY: Kluwer Academic/Plenum Publishers (1999), 179–224.

[15] Kuang D Gu DF Cao H Yuan QF Dong ZX Yu D . Impacts of psychological resilience on self-efficacy and quality of life in patients with diabetic foot ulcers: a prospective cross-sectional study. Ann Palliat Med. (2021) 10:5610–8. doi: 10.21037/apm-21-967, 34044569

[16] Harms CA Cohen L Pooley JA Chambers SK Galvão DA Newton RU. Quality of life and psychological distress in cancer survivors: the role of psycho-social resources for resilience. Psycho-Oncology. (2019) 28:271–7. doi: 10.1002/pon.4934, 30380589

[17] Wang Y Chen Y Xu J Chen H Gao J. Association between resilience and frailty among Chinese older adults. Front Psych. (2022) 13:948958. doi: 10.3389/fpsyt.2022.948958, 35923449 PMC9341387

[18] Lenti MV Brera AS Ballesio A Croce G Padovini L Bertolino G . Resilience is associated with frailty and older age in hospitalised patients. BMC Geriatr. (2022) 22:569. doi: 10.1186/s12877-022-03251-9, 35818046 PMC9275243

[19] Chitalu P Tsui A Searle SD Davis D. Life-space, frailty, and health-related quality of life. BMC Geriatr. (2022) 22:646. doi: 10.1186/s12877-022-03355-2, 35931955 PMC9356461

[20] Lai JC Shui AM Duarte-Rojo A Rahimi RS Ganger DR Verna EC . Association of Frailty with Health-Related Quality of life in liver transplant recipients. JAMA Surg. (2023) 158:130–8. doi: 10.1001/jamasurg.2022.6387, 36515937 PMC9856900

[21] Rakel RE. Depression. Prim Care Clin Off Pract. (1999) 26:211–24. doi: 10.1016/s0095-4543(08)70003-4 10318745

[22] Köhler S Unger T Hoffmann S Mackert A Ross B Fydrich T. The relationship of health-related quality of life and treatment outcome during inpatient treatment of depression. Qual Life Res. (2015) 24:641–9. doi: 10.1007/s11136-014-0811-8 25240747

[23] Schouten RW Harmse VJ Dekker FW van Ballegooijen W Siegert CEH Honig A. Dimensions of depressive symptoms and their association with mortality, hospitalization, and quality of life in Dialysis patients: a cohort study. Psychosom Med. (2019) 81:649–58. doi: 10.1097/PSY.0000000000000723, 31232914 PMC6727937

[24] Lau WKW. The role of resilience in depression and anxiety symptoms: a three-wave cross-lagged study. Stress Health. (2022) 38:804–12. doi: 10.1002/smi.3136, 35191153

[25] Bauer H Emeny RT Baumert J Ladwig KH. Resilience moderates the association between chronic pain and depressive symptoms in the elderly. Eur J Pain (Lond, Engl). (2016) 20:1253–65. doi: 10.1002/ejp.850, 26914727

[26] Wade DT Halligan PW. The biopsychosocial model of illness: a model whose time has come. Clin Rehabil. (2017) 31:995–1004. doi: 10.1177/0269215517709890, 28730890

[27] Deng MG Liu F Liang Y Wang K Nie JQ Liu J. Association between frailty and depression: a bidirectional Mendelian randomization study. Sci Adv. (2023) 9:eadi3902. doi: 10.1126/sciadv.adi3902, 37729413 PMC10511184

[28] Kojima M Kojima T Waguri-Nagaya Y Takahashi N Asai S Sobue Y . Depression, physical function, and disease activity associated with frailty in patients with rheumatoid arthritis. Mod Rheumatol. (2021) 31:979–86. doi: 10.1080/14397595.2020.1838402 33066713

[29] Ali S Stone M Skinner TC Robertson N Davies M Khunti K. The association between depression and health-related quality of life in people with type 2 diabetes: a systematic literature review. Diabetes Metab Res Rev. (2010) 26:75–89. doi: 10.1002/dmrr.1065, 20186998

[30] Kulakçı-Altıntaş H Ayaz-Alkaya S. Health literacy and successful aging in older adults: a predictive correlational design: health literacy and successful aging. Geriatr Nurs (New York, NY). (2024) 61:408–13. 10.1016/j.gerinurse.2024.12.003 39689374

[31] Geschke K Wuttke A. Positive aging-how does it work?: contribution of resilience to the prevention of depressive disorders in old age. Z Gerontol Geriatr. (2024) 57:179–85. 10.1007/s00391-024-02296-2 38634908

[32] Reich AJ Claunch KD Verdeja MA Dungan MT Anderson S Clayton CK . What does "successful aging" mean to you? - systematic review and cross-cultural comparison of lay perspectives of older adults in 13 countries, 2010-2020. J Cross Cult Gerontol. (2020) 35:455–78. doi: 10.1007/s10823-020-09416-6, 33064233

[33] Parsons PA. The ecological stress theory of aging and hormesis: an energetic evolutionary model. Biogerontology. (2007) 8:233–42. doi: 10.1007/s10522-007-9080-z, 17473992

[34] Selvin S. Statistical Analysis of Epidemiologic Data. 3rd ed. New York: Oxford University Press (2004).

[35] Harrison JE Weber S Jakob R Chute CG. ICD-11: an international classification of diseases for the twenty-first century. BMC Med Inform Decis Mak. (2021) 21:206. doi: 10.1186/s12911-021-01534-6, 34753471 PMC8577172

[36] Linn BS Linn MW Gurel L. Cumulative illness rating scale. J Am Geriatr Soc. (1968) 16:622–6. doi: 10.1111/j.1532-5415.1968.tb02103.x 5646906

[37] Ji P Zhang L Gao Z Ji Q Xu J Chen Y . Relationship between self-esteem and quality of life in middle-aged and older patients with chronic diseases: mediating effects of death anxiety. BMC Psychiatry. (2024) 24:7. doi: 10.1186/s12888-023-05459-4, 38166844 PMC10763298

[38] Connor KM Davidson JR. Development of a new resilience scale: the Connor-Davidson resilience scale (CD-RISC). Depress Anxiety. (2003) 18:76–82. doi: 10.1002/da.10113, 12964174

[39] Wang M Zhang L Ma J Sun H Gao Z Hu M . Mediating effect of successful aging on the relationship between psychological resilience and death anxiety among middle-aged and older adults with hypertension. Front Public Health. (2023) 11:1116263. doi: 10.3389/fpubh.2023.1116263, 37808974 PMC10552859

[40] Gobbens RJJ Van Assen MALM Luijkx KG Wijnen-Sponselee MT Schols JMGA. The Tilburg frailty indicator: psychometric properties. J Am Med Dir Assoc. (2010) 11:344–55. doi: 10.1016/j.jamda.2009.11.003 20511102

[41] Xue R Chen B Ma R Zhang Y Zhang K. Association of multidimensional frailty and quality of life in middle-aged and older people with stroke: a cross-sectional study. J Clin Nurs. (2023) 33:1562–70. doi: 10.1111/jocn.16969 38131358

[42] Fairley JL Hansen D Proudman S Sahhar J Ngian GS Walker J . Progression and clinical implications of frailty in patients with systemic sclerosis. Clin Rheumatol. (2024) 44:305–17. doi: 10.1007/s10067-024-07253-3, 39656398

[43] Jiang N Su Y Zhang Y. The association between frailty and depression of older adults with Cancer in China: the mediating effect of social support. Clin Interv Aging. (2024) 19:2059–67. doi: 10.2147/CIA.S487602, 39655270 PMC11626967

[44] Kroenke K Spitzer RL Williams JB. The PHQ-9: validity of a brief depression severity measure. J Gen Intern Med. (2001) 16:606–13. doi: 10.1046/j.1525-1497.2001.016009606.x, 11556941 PMC1495268

[45] Huang H Lu M Zhang P Xiao L Zhang W Xu Y . Association between malnutrition, depression, anxiety and fatigue after stroke in older adults: a cross-lagged panel analysis. Aging Clin Exp Res. (2024) 37:4. doi: 10.1007/s40520-024-02892-7, 39718729 PMC11668841

[46] Lee JE Kahana B Kahana E. Successful aging from the viewpoint of older adults: development of a brief successful aging inventory (SAI). Gerontology. (2017) 63:359–71. doi: 10.1159/000455252, 28297704

[47] Duman İ Kazak Salti A Vefikuluçay Yilmaz D. Factors affecting successful aging of older adults and the relationship between leisure activities duration and successful aging. J Appl Gerontol. (2024) 44:450–7. doi: 10.1177/07334648241273432 39171492

[48] Ware J Jr Kosinski M Keller SD. A 12-item short-form health survey: construction of scales and preliminary tests of reliability and validity. Med Care. (1996) 34:220–33. doi: 10.1097/00005650-199603000-00003, 8628042

[49] Beyer M Bischoff C Lässing J Gockel I Falz R. Effects of postoperative physical exercise rehabilitation on cardiorespiratory fitness, functional capacity and quality of life in patients with colorectal, breast, and prostate cancer - a systematic review and meta-analysis. J Cancer Res Clin Oncol. (2024) 151:13. doi: 10.1007/s00432-024-06064-y, 39718582 PMC11668849

[50] Yadav UN Ghimire S Mehta R Karmacharya I Mistry SK Ali AM . Exploring the pathways between depression, anxiety, stress, and quality of life on life satisfaction: a path analysis approach. BMC Geriatr. (2024) 24:1025. doi: 10.1186/s12877-024-05618-6, 39709336 PMC11662701

[51] Rothman KJ. No adjustments are needed for multiple comparisons. Epidemiology. (1990) 1:43–6. doi: 10.1097/00001648-199001000-00010 2081237

[52] Franjić D Babić D Marijanović I Martinac M. Association between resilience and quality of life in patients with colon cancer. Psychiatr Danub. (2021) 33:297–303. 35150498

[53] Kalisch R Baker DG Basten U Boks MP Bonanno GA Brummelman E . The resilience framework as a strategy to combat stress-related disorders. Nat Hum Behav. (2017) 1:784–90. doi: 10.1038/s41562-017-0200-8, 31024125

[54] Zhang Z Stein KF Norton SA Flannery MA. An analysis and evaluation of Kumpfer's resilience framework. ANS Adv Nurs Sci. (2023) 46:88–100. doi: 10.1097/ans.0000000000000446 36044351

[55] Mei Y Yang X Gui J Li Y Zhang X Wang Y . The relationship between psychological resilience and quality of life among the Chinese diabetes patients: the mediating role of stigma and the moderating role of empowerment. BMC Public Health. (2023) 23:2043. doi: 10.1186/s12889-023-16927-7, 37858079 PMC10585926

[56] Dai W Zeng Y Liang E Zhou Q Zhang L Peng J. The actuality of resilience, social support and quality of life among patients with inflammatory bowel disease in China. Nurs Open. (2022) 9:2190–8. doi: 10.1002/nop2.946, 34037332 PMC9190699

[57] Freidl W Rásky E Stronegger WJ. Operationalisation of a demand/resource model of health: an explorative study. J Epidemiol Community Health. (1999) 53:187–8. doi: 10.1136/jech.53.3.187, 10396497 PMC1756837

[58] Geerinck A Locquet M Bruyère O Reginster JY Beaudart C. Evaluating quality of life in frailty: applicability and clinimetric properties of the SarQoL(®) questionnaire. J Cachexia Sarcopenia Muscle. (2021) 12:319–30. doi: 10.1002/jcsm.12687, 33644998 PMC8061363

[59] Oh GH Yeom CW Son KL Lee KM Kim WH Jung D . Study examines resilience, depression, and quality of life in patients with breast cancer. Saudi Med J. (2021) 42:1258. PMC9149737 34732563

[60] Wierenga KL Lehto RH Given B. Emotion regulation in chronic disease populations: an integrative review. Res Theory Nurs Pract. (2017) 31:247–71. doi: 10.1891/1541-6577.31.3.247, 28793948 PMC5992894

[61] Carvalho IG Bertolli ED Paiva L Rossi LA Dantas RA Pompeo DA. Anxiety, depression, resilience and self-esteem in individuals with cardiovascular diseases. Rev Lat Am Enfermagem. (2016) 24:e2836. doi: 10.1590/1518-8345.1405.2836, 27901221 PMC5172617

[62] Saraçlı Ö Akca AS Atasoy N Önder Ö Şenormancı Ö Kaygisız İ . The relationship between quality of life and cognitive functions, anxiety and depression among hospitalized elderly patients. Clin Psychopharmacol Neurosci. (2015) 13:194–200. doi: 10.9758/cpn.2015.13.2.194, 26243848 PMC4540029

[63] Scheel M Frøslund-Jensen J. Systems theory: stress, adaptation. Sygeplejersken. (1979) suppl) 79:16–21. 257854

[64] Kim GM Lim JY Kim EJ Park SM. Resilience of patients with chronic diseases: a systematic review. Health Soc Care Community. (2019) 27:797–807. doi: 10.1111/hsc.12620, 30027595

[65] Hamaker M Gijzel S Rostoft S van den Bos F. Intrinsic capacity and resilience: taking frailty to the next level. J Geriatr Oncol. (2023) 14:101421. doi: 10.1016/j.jgo.2022.101421, 36657249

[66] Gilmore N Kehoe L Bauer J Xu H Hall B Wells M . The relationship between frailty and emotional health in older patients with advanced cancer. Oncologist. (2021) 26:e2181–91. doi: 10.1002/onco.13975, 34510642 PMC8649062

[67] Das P Naing NN Wan-Arfah N Naing Noor Jan KO Kueh YC Rasalingam K. Depression and quality of life in patients with neurological disorder in a Malaysian hospital. East Asian Arch Psychiatr. (2019) 29:66–70. doi: 10.12809/eaap1777 31237249

[68] da Costa JP Vitorino R Silva GM Vogel C Duarte AC Rocha-Santos T. A synopsis on aging-theories, mechanisms and future prospects. Ageing Res Rev. (2016) 29:90–112. doi: 10.1016/j.arr.2016.06.005, 27353257 PMC5991498

[69] Woo J Leung J Zhang T. Successful aging and frailty: opposite sides of the same coin? J Am Med Dir Assoc. (2016) 17:797–801. doi: 10.1016/j.jamda.2016.04.015, 27236545

[70] Szymkowicz SM Gerlach AR Homiack D Taylor WD. Biological factors influencing depression in later life: role of aging processes and treatment implications. Transl Psychiatry. (2023) 13:160. doi: 10.1038/s41398-023-02464-9, 37160884 PMC10169845

[71] Smith B Shatté A Perlman A Siers M Lynch WD. Improvements in resilience, stress, and somatic symptoms following online resilience training: a dose-response effect. J Occup Environ Med. (2018) 60:1–5. doi: 10.1097/JOM.0000000000001142, 28820863 PMC5770111

[72] Kumar SR Mehta CH Nayak UY. Long-acting formulations: a promising approach for the treatment of chronic diseases. Curr Pharm Des. (2021) 27:876–89. doi: 10.2174/1381612826666200707122012, 32634073

[73] Rubin-Falcone H Weber J Kishon R Ochsner K Delaparte L Doré B . Neural predictors and effects of cognitive behavioral therapy for depression: the role of emotional reactivity and regulation. Psychol Med. (2020) 50:146–60. doi: 10.1017/S0033291718004154, 30739618

[74] Ahmadi KS. The experience of being hospitalized: stress, social support and satisfaction. Int J Nurs Stud. (1985) 22:137–48. doi: 10.1016/0020-7489(85)90024-0, 3847394

[75] Hering A Rendell PG Rose NS Schnitzspahn KM Kliegel M. Prospective memory training in older adults and its relevance for successful aging. Psychol Res. (2014) 78:892–904. doi: 10.1007/s00426-014-0566-4 24744122

[76] Lemire F. Best advice on chronic disease management. Can Fam Physician. (2016) 62:688. PMC4982734 27521401

[77] Powell BJ McMillen JC Proctor EK Carpenter CR Griffey RT Bunger AC . A compilation of strategies for implementing clinical innovations in health and mental health. Med Care Res Rev: MCRR. (2012) 69:123–57. doi: 10.1177/1077558711430690, 22203646 PMC3524416

